# Critical Criteria and Countermeasures for Mobile Health Developers to Ensure Mobile Health Privacy and Security: Mixed Methods Study

**DOI:** 10.2196/39055

**Published:** 2023-03-02

**Authors:** Rita Rezaee, Mahboobeh Khashayar, Saeed Saeedinezhad, Mahdi Nasiri, Sahar Zare

**Affiliations:** 1 Department of Health Information Technology Shiraz University of Medical Sciences Shiraz Iran; 2 Clinical Education Research Center Shiraz University of Medical Sciences Shiraz Iran; 3 Health Human Resources Research Center Shiraz University of Medical Sciences Shiraz Iran; 4 Student Research Committee Shiraz University of Medical Sciences Shiraz Iran; 5 Department of Computer Engineering and Information Technology Shiraz University of Technology Shiraz Iran; 6 Health Information Management Research Center (HIMRC) Kashan University of Medical Sciences Kashan Iran

**Keywords:** telemedicine, mobile apps, privacy, computer security, confidentiality, mHealth, mobile health

## Abstract

**Background:**

Despite the importance of the privacy and confidentiality of patients’ information, mobile health (mHealth) apps can raise the risk of violating users’ privacy and confidentiality. Research has shown that many apps provide an insecure infrastructure and that security is not a priority for developers.

**Objective:**

This study aims to develop and validate a comprehensive tool to be considered by developers for assessing the security and privacy of mHealth apps.

**Methods:**

A literature search was performed to identify papers on app development, and those papers reporting criteria for the security and privacy of mHealth were assessed. The criteria were extracted using content analysis and presented to experts. An expert panel was held for determining the categories and subcategories of the criteria according to meaning, repetition, and overlap; impact scores were also measured. Quantitative and qualitative methods were used for validating the criteria. The validity and reliability of the instrument were calculated to present an assessment instrument.

**Results:**

The search strategy identified 8190 papers, of which 33 (0.4%) were deemed eligible. A total of 218 criteria were extracted based on the literature search; of these, 119 (54.6%) criteria were removed as duplicates and 10 (4.6%) were deemed irrelevant to the security or privacy of mHealth apps. The remaining 89 (40.8%) criteria were presented to the expert panel. After calculating impact scores, the content validity ratio (CVR), and the content validity index (CVI), 63 (70.8%) criteria were confirmed. The mean CVR and CVI of the instrument were 0.72 and 0.86, respectively. The criteria were grouped into 8 categories: authentication and authorization, access management, security, data storage, integrity, encryption and decryption, privacy, and privacy policy content.

**Conclusions:**

The proposed comprehensive criteria can be used as a guide for app designers, developers, and even researchers. The criteria and the countermeasures presented in this study can be considered to improve the privacy and security of mHealth apps before releasing the apps into the market. Regulators are recommended to consider an established standard using such criteria for the accreditation process, since the available self-certification of developers is not reliable enough.

## Introduction

More than 5.19 billion people now use mobile phones, which indicates that mobile phones form an important part of daily life worldwide [1]. Mobile phone features, including mobility, instantaneous availability, and direct communication, have changed the provision of health care services. These features introduce mobile health (mHealth). Of about 2 million smartphone apps available in app stores, 318,000 are health apps [2]. According to a World Health Organization report [3], the penetration of mHealth, with promising results, in low- and middle-income countries would be even more.

mHealth has improved the patient care status through the provision of health care anytime and anywhere [4]. Even in recent years, the integration of mHealth and wireless technologies has provided clinicians with an opportunity to collect real-time data via wearable sensors [5]. Health information is deemed sensitive, and its protection is of significance. Nevertheless, smartphones are vulnerable to a wide range of security threats [6]. Moreover, electronic transmission of information has brought about concerns about its privacy and security. A national survey showed that 1 of the common reasons for people not having downloaded health apps is concern about apps gathering their data [7,8]. The privacy and confidentiality of information, as a human right, have long been considered in law and regulations. Well-known examples are the Health Insurance Portability and Accountability Act (HIPAA) rules, the General Data Protection Regulation (GDPR), and the Common Rule [9-11]. The terms “security,” “privacy” and “confidentiality” are all separate yet connected concepts that need to be addressed. The National Committee for Vital and Health Statistics [12] defines and distinguishes these concepts as follows:

Health information privacy is an individual’s right to control the acquisition, uses, or disclosures of his or her identifiable health data. Confidentiality, which is closely related, refers to the obligations of those who receive information to respect the privacy interests of those to whom the data relate. Security is altogether different. It refers to physical, technological, or administrative safeguards or tools used to protect identifiable health data from unwarranted access or disclosure.

Despite the importance of the privacy and confidentiality of patients’ information, studies report that mHealth apps may share the information with third parties, which raises the risk of violating patients’ privacy and confidentiality [13-15]. Dehling et al [16] evaluated the information security and privacy of 24,405 health-related apps and revealed that most apps request access to sensitive information. Robillard et al [17] reported that most of the apps do not include privacy policies and terms of the agreement. Moreover, it has been shown that many apps provide an insecure infrastructure and security is not a priority for the developers [18]. Similar studies emphasize assessing mHealth apps for the privacy, security, and confidentiality of information to minimize the associated risks [16,19,20].

Criteria have been proposed in previous studies for assessing mHealth apps. Benjumea et al [21] proposed a novel scale to assess the privacy policy of mHealth apps. However, the scale considers only specific items associated with the privacy policy content based on the GDPR rather than considering security and privacy in general. Another study [22] also proposed a heuristic evaluation approach to assessing the privacy of mHealth apps, but that is a time-consuming approach because heuristics require a close reading of the privacy policy. Another study proposed a security-testing method for Android mHealth apps designed based on a threat analysis, considering probable attack scenarios and vulnerabilities associated with the domain [18]. They assessed security using novel dynamic and static analysis testing methods that were expensive to perform. Benjumea et al [23] conducted a scoping review on studies exploring privacy issues in mHealth apps. Finding that most studies assess the apps based on heterogeneous criteria, Benjumea et al [23] emphasized the importance of developing a scale based on more objective criteria for evaluating privacy issues. In addition, the mHealth field faces a variety of legal and cultural differences over privacy between nations, so it needs a comprehensive tool for assessing both privacy and security issues [24]. Thus, developing a comprehensive tool assessing both privacy and security sounds necessary. This study aims to develop and validate a comprehensive tool to be considered by developers for assessing both the security and the privacy of mHealth apps targeting patients.

## Methods

### Study Design

This study was conducted to answer the following question: What security and privacy criteria should be considered when developing or assessing mHealth apps targeting patients based on 3 main phases: item generation, tool development, and tool evaluation? These main phases [25] were performed based on 4 steps: (1) identifying criteria associated with mHealth apps’ security/privacy according to a literature search (item generation); (2) conducting an expert panel for determining the categories and subcategories according to meaning, repetition, and overlap (tool development); (3) testing the validity of the instrument (tool evaluation); and (4) testing the reliability of the instrument (tool evaluation).

#### Stage 1: Literature Review

An unstructured literature search was performed to identify papers on app development, assessment, security, or privacy that reported criteria for the security and privacy of mHealth. PubMed, Scopus, Web of Science, and Cochrane were searched for English language papers published until December 15, 2021, without a time limitation. The search strategy (Multimedia Appendix 1) included a combination of 4 keywords: (“mobile device” OR “mobile phone” OR smartphone OR “smart Phone” OR mHealth OR “mobile health”) AND (App OR apps OR application*) AND (security OR privacy OR confidentiality OR cybersecurity) AND (guideline* OR standard* OR criteria OR risk* OR assess* OR evaluat* OR measure).

The HIPAA and GDPR websites were searched for relevant criteria. After removing duplicate papers, the titles and abstracts of the studies were screened for inclusion. The full text of potentially relevant papers was investigated based on study objectives. Studies substantially focusing on security or privacy, not just mentioning them in passing, and stating clear criteria for assessing the privacy/security of mHealth apps were included. Studies evaluating the privacy or security of mHealth apps were also included to specify the criteria used for evaluation. Papers proposing a secure architecture, investigating technical solutions for mHealth apps (eg, access control, authentication approaches, encryption methods), presenting technical solutions for connecting mHealth apps to cloud computing or the internet of things devices or conducted on wearable devices without connecting to a mobile device, and discussing mobile phone access to electronic health records were excluded. Papers focusing on mHealth apps targeting users other than patients, focusing on app quality or determining functional requirements, and examining user experiences were also excluded. The criteria were extracted using content analysis.

#### Stage 2: Expert Panel

The list of primary criteria extracted through the literature search was presented to a focus group including 2 health information technology (HIT) specialists, 2 medical informatics specialists, and 1 software and IT specialist. The focus group discussion consisted of 4 major steps: designing research, collecting data, analyzing, and reporting results through a moderated interaction [26]. The experts discussed and categorized the criteria and decided over their inclusion or exclusion based on the relevancy, clarity, importance, comprehensiveness, and overlap with other included criteria, and they determined subcategories based on meaning, repetition, and overlap. This method can have a high level of validity due to the interaction among experts that confirms, reinforces, or rejects the individual respondents’ contributions. The criteria extracted through the focus group discussion were used in the next stage.

#### Stage 3: Testing the Validity of the Instrument

Quantitative and qualitative methods were used for validating the instrument. To validate the instrument based on the qualitative approach, face validity was checked through face-to-face interviews by 8 HIT specialists and 5 software and IT experts. The inclusion criteria for the experts included specialists in HIT, IT, or software, with a master’s degree in science or higher, with at least 1-year work experience in software security, network security, health information security, or mobile app development. The criteria were modified based on the experts’ comments.

To validate the instrument quantitatively, the impact score was calculated for each criterion. The impact score determines inappropriate criteria. Thus, the criteria were evaluated based on a 5-point Likert scale ranging from 5 (very important) to 1 (not at all important). The impact score for each criterion was calculated as follows:

Impact score = Frequency (%) × Importance

Content validity was evaluated by 16 other IT (n=8, 50%) and software (n=8, 50%) experts, of whom 3 (18.8%) experts did not participate. Thus, to make sure the most essential criteria for the study objective were chosen, the content validity ratio (CVR) was measured. The CVR was calculated based on the following formula:



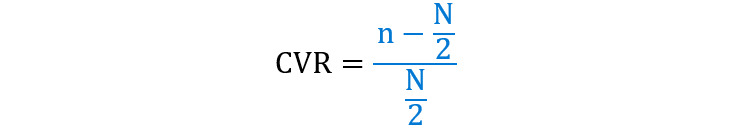



According to the Lawshe table, if the number of experts in the panel is 13, the minimal acceptable CVR is 0.54.

In addition, to ensure the relevancy and clarity of each criterion, the content validity index (CVI) was measured. Thus, the 13 experts also completed a 4-point scale based on relevance, clarity, and simplicity for the criteria. The CVI was calculated using the following formula:



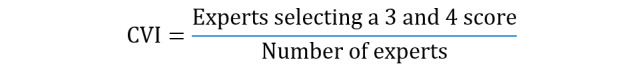



The criteria were included in the final assessment tool if the CVI was ≥0.79 [27,28]. If the CVI was between 0.70 and 0.79, it needed to be calculated after the criteria were revised by the experts. Criteria with a CVI of <0.70 were removed.

#### Stage 4: Testing Reliability

To assess the reliability of the final tool, the hypertensive self-care app developed in our previous study [29] was selected. The app needs to record a variety of personal information. In total, 30 experts in HIT, medical informatics, IT, and software assessed the reliability of the instrument. The instrument was distributed among these experts twice in a 2-month interval. They were asked to assess the privacy and security of the self-care app using the criteria provided in the checklist. After collecting expert opinions about the self-care app, the data were analyzed using the Cronbach α.

### Ethical Considerations

The research was conducted according to the principles stated by the Vice-Chancellorship for Research Affairs of Shiraz University of Medical Science and approved by the Ethics Review Board of the Vice-Chancellorship for Research Affairs of Shiraz University of Medical Science (ethical code IR.SUMS.REC.1397.500).

## Results

### Study Selection

**Figure 1:**
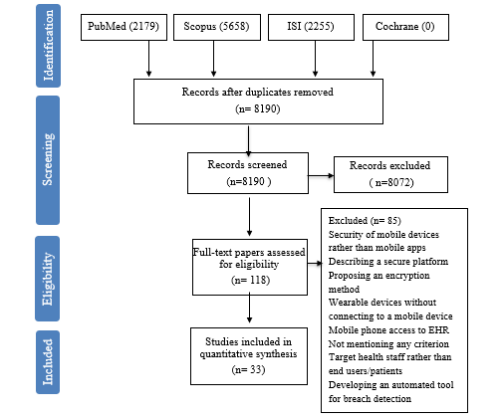
Flow diagram of study selection. EHR: electronic health record.

The search strategy retrieved 10,092 papers, of which 1902 (18.8%) were duplicates. Of the 8190 (81.2%) remaining papers, 8072 (98.6%) were irrelevant. To retrieve the greatest number of possible relevant papers, our search strategy included smartphone or mobile devices as a synonym for mHealth (“mobile device” OR “mobile phone” OR smartphone OR “smart Phone” OR mHealth OR “mobile health”); this resulted in retrieving papers basically irrelevant to the health discipline, in addition to those relevant to the health discipline—for example, studies associated with payment/banking/commercial apps were also retrieved in the primary result. In total, 33 (0.4%) studies were deemed eligible for inclusion in the research (Figure 1). The characteristics of the included studies [13,14,16,18-20,24,30-56] are presented in Multimedia Appendix 2.

**Figure 2:**
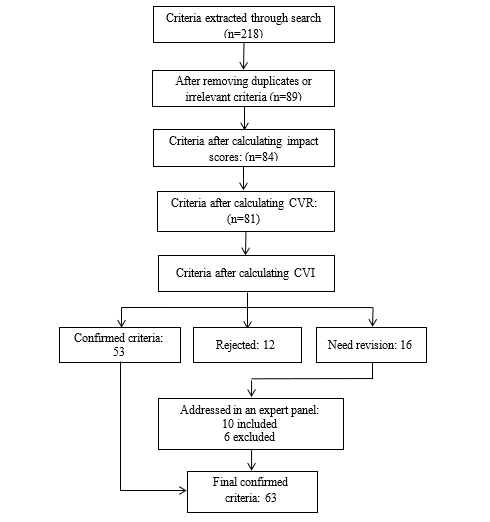
Flowchart of criteria determination. CVI: content validity index; CVR: content validity ratio.

A total of 218 criteria were extracted based on the literature search; of these, 119 (54.6%) were removed as duplicates (showing the same idea) and 10 (4.6%) were deemed irrelevant to the security or privacy of mHealth apps. The remaining 89 (40.8%) criteria were presented to the expert panel. As shown in Figure 2, 63 (70.8%) criteria were confirmed at last.

The mean CVR of the total instrument was 0.72, while the mean CVI was 0.86. Multimedia Appendix 3 shows the complete list of removed criteria in the different phases of the study.

Finally, to measure the reliability of the instrument, the experts were asked to assess the hypertensive self-care app using the instrument. When measuring the reliability of the instrument, 18 (28.6%) of the 63 criteria received the lowest and the highest score of the Likert spectrum (“not at all” and “completely”) equally. Since the variance of equal data was 0, these 18 criteria did not automatically enter for calculating the Cronbach α value. Thus, the test was performed with 45 (71.4%) criteria. The Cronbach α value was 0.89.

The 63 criteria were grouped into 8 categories: authentication and authorization (n=8, 12.7%), access management (n=6, 9.5%), security (n=13, 20.6%), data storage (n=4, 6.3%), integrity (n=2, 3.2%), encryption and decryption (n=5, 9.5%), privacy policy (n=15, 23.8%), and privacy policy content (n=10, 15.9%); see Textbox 1.

**Final privacy and security assessment criteria.**

**1. Authentication and authorization**

1.1. Is there any registration/log-in available in the app?

1.2. Does the app capture a unique username or “fixed device identifier” used as a user identifier (for both patient and health care provider)?

1.3. Are there procedures to verify that any person or entity claiming access to electronic protected health information complies with its claim?

1.4. Are there any ways to monitor the log and report errors?

1.5. Are there any steps to create, change, and protect the password?

1.6. Are the passwords complex enough (ie, of a minimum length, alphanumeric with upper- and lowercase letters and symbols)?

1.7. Are the passwords updated periodically?

1.8. Is the user’s account locked after a determined number of consecutive unsuccessful log-in attempts?

**2. Access management**

2.1. Is there patient-centric access control?

2.2. Are there measures taken to access the health information needed in an emergency?

2.3. Is the user allowed to access personal information and to participate in treatment?

2.4. Does the app facilitate the provision of an electronic copy of data?

2.5. Is the app capable of cutting off or blocking a person's access at any time?

2.6. Are users allowed to control the access level of their health information by third parties?

**3. Security**

3.1. Does the app use secure connections (Secure Socket Layer [SSL]/Transport Layer Security [TLS])?

3.2. Can the data be remotely controlled if the mobile phone is lost/stolen?

3.3. Does the app use a secure platform for transmitting health data?

3.4. Does the app protect network traffic by strong coding?

3.5. Are default measures present to protect against, identify, and report security incidents/malware?

3.6. Does the app use external devices?

3.7. Does the app use random number generators?

3.8. Are users able to change individual profiles according to the policy of the mobile health (mHealth) app?

3.9. Does the app require interaction with the user while performing a sensitive operation or communicating with an untrusted app?

3.10. Does the app use cookies?

3.11. Is the security policy transparent and easy to find?

3.12. Are there reminders for periodic system security updates?

3.13. Has anyone been appointed to assume security responsibility?

**4. Data storage**

4.1. Are data stored locally on the device? If no, are the users notified about using another platform for storing their data?

4.2. Are data centers in a secure condition?

4.3. Are data stored on the mobile phone or to the app company’s own servers?

4.4. Are there any steps to recover lost data or any backup?

**5. Integrity**

5.1. Are there electronic mechanisms to verify that health information is not unauthorized, altered, or destroyed (eg, check-sum verification or digital signatures)?

5.2. Are security measures in place to prevent the unauthorized destruction or tampering of health information that is being exchanged electronically?

**6. Encryption and decryption**

6.1. Does the app use a strong modern encryption/decryption mechanism?

6.2. Is a proper method of encryption selected and implemented (eg, use encryption through https rather than http)?

6.3. Are the data stored encrypted?

6.4. Are the data transmitted encrypted?

6.5. Is the username/password/keys encrypted?

**7. Privacy**

7.1. Is there a privacy policy on the app or a link to the full privacy policy?

7.2. Are there any restrictions on the use or disclosure of information contained in the app?

7.3. Are there restrictions on the collection of information?

7.4. Does the app have the ability to disclose information on social media by the user?

7.5. Has the principle of protecting the confidentiality of data been met?

7.6. Does the app state which regulation it complies with and which country the regulation belongs to?

7.7. Does the app ask normal permissions and provide justification for that?

7.8. Is identifiable information anonymized and de-identifiable? If anonymization is not possible, are users informed?

7.9. Have any measures been taken to notify the users of their privacy rights?

7.10. Will the user be informed of any leaks or breaches?

7.11. Does the app have the ability to manage alerts (eg, hide them from the lock screen)?

7.12. Is the privacy policy easy to find, clear, readable, and up to date?

7.13. Are users able to manipulate or completely delete personal profiles and any data archives?

7.14. Are users informed about any security or privacy measures?

7.15. Does the app prevent disclosure of data about the location or sensor type of the user?

**8. Privacy policy content**

8.1. Is there a time limit for data retention?

8.2. Is the content of the contract with third parties clearly stated?

8.3. Does the app mention the collection of user data and how they are being used?

8.4. Does the privacy policy describe the purpose and the type of information collected?

8.5. Is the data ownership specified?

8.6. Are the administrative details stated (identify data controller or responsible legal entity, legal jurisdiction governing policy, jurisdictions under which transmitted data will be processed, date of policy and next review)?

8.7. Is there an explanation about the retention policy for the health information?

8.8. Does the privacy policy explain the manipulation of data by the developer or third parties?

8.9. Does the privacy policy explain the complaints procedures?

8.10. Does the privacy policy explain the procedures for changing the terms of the policy?

## Discussion

### Principal Findings

In this study, we developed an instrument for assessing the security and privacy of mHealth apps. The criteria proposed in this tool were classified into 8 categories: authentication and authorization, access management, security, data storage, integrity, encryption and decryption, privacy, and privacy policy. These criteria can be considered by mHealth app developers to improve the privacy and security of their apps before releasing them into the market.

### Authentication and Authorization

The criteria in the tool suggest implementing rigorous authentication and authorization techniques. More time and effort should be devoted to preventing unauthorized access to personal health information. The developers are asked to provide a unique master ID and a secret key identity for users to control role-based access and verify users’ activities according to the defined identity and roles. Authentication via a fingerprint or a personal identification number is necessary for internal storage, internal cache, external storage, and databases [57]. Audit trails should be in place to track logs, protect data, and identify which user’s health data was handled and by whom. Each user should be able to create, change, and protect their passwords. The developers should make sure the passwords are strong enough and are changed periodically, because there are tools that produce 10^14^ guesses in an hour to find the correct password [58]. There are some strategies to be used by developers to make sure passwords are secure; these include enforcing password complexity; making passwords unviewable, even to the app administrator; and locking a user’s account after a determined number of consecutive unsuccessful log-in attempts. System-generated passwords can be strong, but they do not guarantee memorability. Using Optiwords8 passwords [59], based on the picture superiority effect on the mobile phone keyboards, guarantees the security of passwords, while keeping them usable and memorable as a result.

### Access Management

mHealth app developers need to define access controls for their team members as well as users. For those apps providing health care provider–patient communication, granting access to specific app functions should be based on predetermined and confirmed roles and attributes. Patients should be users allowed to control the access level of their health information by third parties. Greene et al [60] proposed the ShareHealth framework, which provides cryptographically enforced access to data. The framework takes advantage of combining a robust cryptographic scheme, hash chains (to control access by data time), and attribute-based encryption (to control access by data type). Rectification, deleting, or blocking of data should be facilitated for users [53].

### Security

Some mHealth apps use connections for several purposes, including fetching mail, sending analytics data, or checking for updates. To protect the authenticity, confidentiality, and integrity of the connection, developers are encouraged to use an up-to-date version of the Transport Layer Security protocol and its predecessor, the Secure Socket Layer (SSL) [54]. SSL protocols provide an encrypted link that connects a server and a client and makes sure the transmitted data remain impossible to read and are kept private; however, if the coding is not strong enough, hackers would be able to interpret health data during transmission [44]. There should be a functionality of remote control of data to securely transfer, retrieve, or completely erase health information if the mobile phone is stolen/lost [35]. However, it is safer to store data on users’ own devices rather than on the app company’s servers [13]. Some apps use external devices, such as cameras, sensors, or payment apps, to improve their functionality, but this endangers users’ confidentiality through attacks, such as external-device misbonding [48]. Moreover, using cookies can jeopardize user privacy especially those used for data analysis by third parties [14]. Users should be able to manipulate their profile or delete it completely when they stop using an app [31].

### Encryption and Decryption

Bhanot and Hans [61] compared various encryption algorithms based on different criteria, such as cryptography type, key management, keys number, and bit numbers used in a key. They found that elliptic-curve cryptography and blowfish encryption algorithms are the best, providing higher security levels as well as faster encryption speeds, which is required for mobile devices due to less power consumption [61]. Security measures, such as wired equivalent privacy, which is used to provide security to mobile devices, are vulnerable to hackers [62,63]. Thus, developers are required to perform a security risk analysis to determine vulnerabilities at each stage of design and implementation throughout testing and use. Arora et al [64] suggest using a “red team” for risk analysis. Red team experts are charged with hacking cyber systems in order to detect weaknesses.

### Privacy

Papageorgiou et al [49] found that although many of the studied apps ask for dangerous permissions (eg, read/write external storage, access camera, location, and contacts), they do not follow well-known regulations, such as HIPAA. Developers are required to collect data as much as they need to provide their services, so they are required to provide reasons for permissions they ask for, the type of data they collect, and how the data will be used by them or third parties, including insurance companies, government institutions, or even research centers [18,38]. Third-party usage of health data can bring about privacy intrusions, such as loss of insurance coverage or higher insurance premiums [65]. Complying with regulations and which country these regulations belong to is also important because when enforcing privacy rights, the regulations may differ from the users’ own country [13]. Users’ records should be stored in incognito forms, which are anonymized and unidentifiable; if anonymization is not possible, users should be informed [40].

All mHealth apps need to provide a transparent, precise, and well-readable privacy policy statement or a link to the complete privacy policy. Procedures for refusing data sharing, consequences of not providing/sharing data, procedures for changing the terms of the policy, procedures for editing or deleting data held by developers/third parties, procedures for complaints, and procedures for handling data for vulnerable users are subsets of “user rights” a privacy policy should contain. In addition, a data retention policy, data ownership, date of the policy, and next reviews should be contained as “administrative details” of the privacy policy. Users' access to their health information is another right. A systematic review [66] indicated that patients’ access to their health information has a positive impact. A similar study [21] proposed a 14-criteria scale for assessment of a privacy policy based on the GDPR. Although the items by proposed Benjumea et al [21] overlap our proposed criteria (some with different words but similar concepts), they include 5 items not included in our tool; 2 items are “legal basis for processing” and “legitimate interests from controller” that imply the bases for the processing determined by the GDPR. This may be similar to the criteria associated with permission/consent and how users’ data will be processed/used, which are considered in our tool in general. Another item is “transfers to non-EU countries,” which sounds similar to the “regulation the mHealth app comply with and the country (as general, not only European ones) that the regulation belongs to” also considered in our tool. The fourth item is “obligation to provide personal data,” which can be considered as a subset of “user rights” [34] (existent among our criteria). As mentioned earlier, users need to be informed about the consequences of not providing their information. The last item is “existence of automated decision-making or profiling,” which is not included in our tool. It also worth to note that the criteria proposed in our study are general criteria for assessing both privacy and security classified into 8 categories. We tried to determine a comprehensive list of criteria, but we also faced a restriction to limit our criteria to general important aspects of privacy and security, because including a large number of criteria makes it difficult for assessors to consider all of them and this may result in rejection of the tool. That is why we tried to use general concepts that cover more specific criteria (eg, user rights) or merge some criteria into a single one (eg, administrative details).

### Limitations

In this study, a list of criteria was proposed using published papers. A limitation of this study is conducting an unstructured literature search, due to which we missed some related papers. However, to the best of our knowledge, many of the criteria included in our study overlap those that were not included. Another limitation is the large number of included criteria, which may make it difficult for assessors to consider all of them; however, we tried to limit our criteria to important ones to make them more applicable, and we also used general concepts that cover more specific criteria (eg, user rights) or merged some items into a single one (eg, administrative details). Another limitation is the difficulty in assessing some criteria—for example, app compliance with regulations may not be clearly stated in the app. It is recommended that future studies verify the proposed criteria using mobile apps. However, they should be considered in conjunction with other assessment strategies, such as risk analysis, data leakage detection, and continuous revision accordingly. Moreover, this study focused on the security and privacy challenges of mHealth apps, but there are other important challenges, such as interoperability. Thus, it is recommended that future studies combine both aspects to obtain not only a secure system but also an interoperable one, because mHealth apps communicate with a variety of sources.

### Conclusion

With the evolution in the health field through smartphones and mHealth apps, privacy and security challenges need to be addressed. The proposed comprehensive criteria can be used as a quick guide for app designers, developers, regulators, and even researchers. The criteria and the countermeasures presented in this study can be considered to improve the privacy and security of an mHealth app before releasing it into the market. Regulators are recommended to consider an established standard using such criteria for the accreditation process, since the available self-certification of developers is not reliable enough.

## Appendix

Multimedia Appendix 1 This is the search strategy.

Multimedia Appendix 2 Characteristics of the included studies.

Multimedia Appendix 3 The complete list of removed criteria.

## Abbreviations

CVI: content validity index
CVR: content validity ratio
GDPR: General Data Protection Regulation
HIPAA: Health Insurance Portability and Accountability Act
HIT: health information technology
mHealth: mobile health
