# Supplementary material for: Critical Criteria and Countermeasures for Mobile Health Developers to Ensure Mobile Health Privacy and Security: Mixed Methods Study
Source: JMIR Mhealth Uhealth. 2023 Mar 2;11:e39055. doi: 10.2196/39055 (PMC10020905; doi:10.2196/39055)
Supplement: Multimedia Appendix 1 [file mhealth_v11i1e39055_app1.docx]

PubMed: 15 december 2021 : 2179 (after English filter)

((telephone[Title/Abstract] OR mobile[Title/Abstract] OR phone[Title/Abstract] OR smartphone[Title/Abstract] OR smart-phone[Title/Abstract] OR mhealth[Title/Abstract] OR m-health[Title/Abstract] OR "m health"[Title/Abstract] OR e-health[Title/Abstract] OR ehealth[Title/Abstract] OR "e health"[Title/Abstract] OR telecare[Title/Abstract] OR tele-care[Title/Abstract] OR telemedicine[Title/Abstract] OR telemed[Title/Abstract] OR telehealth[Title/Abstract] OR tele-health[Title/Abstract] OR telenursing[Title/Abstract] OR tele-nursing[Title/Abstract] OR telemedicine[MeSH Terms] OR telephone[MeSH Terms] OR “Computers, Handheld”[MeSH Terms]) AND (app[Title/Abstract] OR apps[Title/Abstract] OR application*[Title/Abstract] OR “Mobile Applications”[MeSH Terms]) AND (privacy[Title/Abstract] OR confidential*[Title/Abstract] OR secur*[Title/Abstract] OR bioethic*[Title/Abstract] OR ethic*[Title/Abstract] OR consent*[Title/Abstract] OR "e-consent*"[Title/Abstract] OR "data protection"[Title/Abstract] OR cybersecurity[Title/Abstract] OR “cyber security”[Title/Abstract] OR privacy[MeSH Terms] OR confidentiality[MeSH Terms] OR "Computer Security"[MeSH Terms]) AND (guideline*[Title/Abstract] OR standard*[Title/Abstract] OR criteria[Title/Abstract] OR risk*[Title/Abstract] OR assess*[Title/Abstract] OR evaluat*[Title/Abstract] OR measure[Title/Abstract]))

Scopus: 15 december 2021 : 5658 (after English filter)

TITLE-ABS KEY ("mobile device" OR "mobile phone" OR smartphone OR "smart Phone" OR mhealth OR “m-health” OR “m health”OR "mobile health") AND (App OR apps OR application* OR “Mobile Application*”) AND (security OR privacy OR confidentiality OR ethic* OR “data protection” OR consent* OR “e-consent*” OR cybersecurity OR “cyber security”) AND (guideline* OR standard* OR criteria OR risk* OR assess* OR evaluat* OR measure)

Web of science: 15 december 2021 : 2255 (after English filter)

(("mobile device" OR "mobile phone" OR smartphone OR "smart Phone" OR mhealth OR “m-health” OR “m health”OR "mobile health") AND (App OR apps OR application* OR “Mobile Application*”) AND (security OR privacy OR confidentiality OR ethic* OR “data protection” OR consent* OR “e-consent*” OR cybersecurity OR “cyber security”) AND (guideline* OR standard* OR criteria OR risk* OR assess* OR evaluat* OR measure))

Cochrane 0 result

(("mobile device" OR "mobile phone" OR smartphone OR "smart Phone" OR mhealth OR “m-health” OR “m health”OR "mobile health") AND (App OR apps OR application* OR “Mobile Application*”) AND (security OR privacy OR confidentiality OR ethic* OR “data protection” OR consent* OR “e-consent*” OR cybersecurity OR “cyber security”) AND (guideline* OR standard* OR criteria OR risk* OR assess* OR evaluat* OR measure))
