# Supplementary material for: Critical Criteria and Countermeasures for Mobile Health Developers to Ensure Mobile Health Privacy and Security: Mixed Methods Study
Source: JMIR Mhealth Uhealth. 2023 Mar 2;11:e39055. doi: 10.2196/39055 (PMC10020905; doi:10.2196/39055)
Supplement: Multimedia Appendix 2 [file mhealth_v11i1e39055_app2.docx]

**Multimedia appendix 2: The characteristics of the included studies**

| **Paper** | **Publication Year** | **Objective** | **Type of study** | **Subject category** | **Short study description** | **Criteria** | **Description** |
| --- | --- | --- | --- | --- | --- | --- | --- |
| Jones et al. (30) | 2011 | To discuss the security, privacy and interoperability of mobile health  applications | Mhealth Apps assessment | Health and wellbeing apps | A “Consumer Perspective Framework” including “data issues” and “software issues” is developed. | Data issues:  1-Storage and Privacy  2-Ownership  3-Corporate Use  4-Location  Software issues:  1-Accessibility  2-Clinical effectiveness  3-Credibility  4-Information quality  5-Consumer usage | 1-Secure personally identifiable health information, strong encryption and authentication  2- Licensing agreement cover data storage, use (primary and  secondary), durability and ownership  3- Secondary use of data should be clearly understood through licensing agreement  4- Where the data is processed or stored  1-Easily find, select, and download an app  2-Describing the app purpose and clinical quality  3- Recognize credible and safe apps  4- Quality and accuracy of apps information  5- Motivations behind the use of an app |
| Adhikari et al. (31)  (Australia) | 2014 | To investigate the strengths and limitations of data privacy and security | Mhealth Apps assessment | General | Through a systematic literature review and a comparative analysis of the 20 most popular mHealth apps, a set of risk and safe features were identified. | Security issues  1-User registration  2-Update personal profiles  3-User authentication  Privacy issues  1-Delete any personal information completely  2-Data storage  3-Share data with a third party  4-Security explained  5- Privacy policy (PP) | 1-Providing detailed information may result in compromising data privacy.  2-change individual profiles according to the policy of the mHealth apps.  3-User name and password prevent unauthorized access  1-Users need to be able to delete their personal profile and any data archives when they stop using an app  2-data can be stored locally on a device or in a cloud  3-sharing users’ data to generate revenue or cost, endangers privacy and security  4-User informed about any data privacy and security measures  5-Existence of PP |
| Rowan et al. (32) (USA) | 2014 | To compare privacy policy including application permissions requested and several readability metrics of PP | Mhealth Apps assessment | Health and fitness apps | 20 popular health and fitness app that collect data were compared regarding the required permissions and readability test | 1-Required permissions  2-Readability | 1-reduce the required permissions  2-clearly describe how users’ personal information is collected, stored, and transferred, mobile-friendly short forms with links to their full PP, use standards for language, terminology, and PP formats to lower the users cognitive load, use seals of accreditation within pp to inform users of their trustworthiness and healthcare quality |
| Carter et al. (33) (Australia) | 2015 | To present ethical guidelines and future directions of mobile phones | Viewpoint paper | Parkinson’s disease apps | Ethical issues raised by the use of mobile phones for Parkinson’s disease are discussed. | 1-Anonymity and de-identification  2- Share data with a third party  3- Data storage and transmission  4- informed consent  5- Clinical effectiveness  6- Accessibility | 1-Identifiable information should be anonymized and deidentifiable, if not possible, developers must inform users.  2-users informed about the risk of third parties accessing data, either via hacking, legal interception, incidental discovery by someone accessing the phone, or by telecommunication companies (eg, ISP provider, Google) that may claim ownership of the data recorded by or transmitted through their networks  3- minimize the risk of privacy violations by consideration of how the data are stored locally on the phone or transmitted  4-users informed about the risks and benefits of using mHealth app, understand these risks and make a free decision  5- providing clinically relevant information based on a qualified health care professional or strong empirical evidence |
| Dehling et al. (16) | 2015 | to establish an overview of mHealth apps and Android with a special focus on potential damage to users through information security and privacy infringement | Mhealth Apps assessment | Medical and Health & fitness apps | Apps were clustered to group similar apps by app tagging which created a machine-readable description of app functionality. Subsequently, information security and privacy implications were assessed. | 1-Specificity  2- Leaks  3- Change  4- Loss  5- Value | 1-Health specificity of information available to apps  2-Potential damage through leaks of information (eg, embarrassment, lessened employment prospects)  3- Potential damage through manipulation (change) of information (eg, treatment errors)  4- Potential damage through loss of information (eg, loss of information important for treatment)  5- Value of information to third parties (eg, medical identity theft, selection of employees) |
| Huckvale et al. (34) (UK) | 2015 | To assess the extent to which already-certified apps complied with data  protection principles | Mhealth Apps assessment | General | App assessment | Security:  1- encryption username/ password  2- encryption data storage  3- encryption personal or sensitive information  4- transport layer protection  5-User authentication  6-server-side controls  7-PP topics coverage:  7-1-Uses of data  7-2-Technical concerns  7-3-User rights  7-4-Administrative details | 4- encryption of sensitive information sent 5-capture username/password and fixed device identifier used as user identifier  6- encrypted access to server-side API and authorized access to user data  7-1-Primary/secondary uses of data, sending data to developer-provided online services, advertisers/marketers, analytics/research, while loading content, and anonymous uses  7-2- Technical and procedural security arrangements, how long data will be retained, Inherent risks or limitations of security /The use of cookies  7-3- Procedures for opting out of data sharing, Consequences of not providing or sharing data, Procedures for subject access requests, Procedures for editing or deleting data held by developers/third parties, Complaints procedures, Special procedures for handling data for vulnerable users  7-4- identify data controller or responsible legal entity, Legal jurisdiction governing policy, Jurisdictions under which transmitted data will be processed, Date of policy and next review, Procedures for changing the terms of the policy |
| Knorr and Aspinall (18) | 2015 | To propose a testing method  for Android mHealth apps | Propose a testing method | Hypertension and diabetes apps | A testing method is developed for Android mHealth apps using a threat analysis, considering possible attack scenarios and vulnerabilities, discovering a number of serious vulnerabilities in the most popular applications. 150 apps were assessed using this method and results are presented in another paper which is excluded due to the criteria overlap. | Security:  1-Static code analysis  2-Dynamic analysis  3-Web server connection  4-PP topics coverage:  4-1-Basic PP information  4-2- Accountability  4-3- Security safeguards  4-4-Openness  4-5-Purpose specification  4-6-Individual participation  4-7-Invasiveness | 1-Proper SSL usage, Debug flag, access control, Use of encryption, certificates which sign apps, Code quality, limit Add-ons, and Use malware and privacy scanners  2- Input validation, unencrypted data stored or transmitted, a feature to erase all stored medical data, Reasonable permissions, Secure backup and logging  3- secure web server connection (https), authentication (strong password)  4-1-URL to PP, number of words (indicating coverage), version and country of origin  4-2- present data controller name or detailed contact data  4-3-Describe safeguards  4-4- Describe types of data collected  4-5-Describe the purpose and usage of data  4-6-Describe rights of the individual  4-7-use data for other purposes, store or shared data by a third party |
| Kramer et al. (35) | 2015 | To provide an overview of some regulations and standards in place. | Viewpoint paper | Mental health apps | They reviewed some of the primary legal and regulatory challenges and then provided some practical recommendations. | 1-Secure transmission  2-Encryption  3-Remote control of data  4-Data retention  5-FDA regulation | 1-use current security standards such as Wi-Fi Protected Access for secure transmission  2-Electronic data encryption for transmission using HIPAA compliant encryptions features on one’s smartphone  3- Remote control of data helps securely transfer and store PHI if the mobile is stolen/lost.  4- A lifespan should be considered for the Protected Health Information (PHI) to help delete PHI at the correct time automatically.  5-any tele mental health practitioner who may wish to use mobile apps in their practice with patients should become familiar with FDA regulatory guidance. |
| Martinez-Perez (24) | 2015 | To presents a study of security  and privacy in mHealth | Review | General | A study of the existing laws regulating these aspects in the European Union and the United States, a review of the academic literature  related to this topic, and some recommendations  for designers | 1- Access control  2-Authentication  3- Security and confidentiality  4- Integrity  privacy policies  5- users’ consent  6- Data retention  7- Breach notification | 1- The access control to the PHI must be patient-centric.  2- The authentication must be done with a unique ID and a password only known by the user  3-develop a good strategy to secure  PHI accessed, stored, or transmitted; PHI should be encrypted.  4- a symmetric key-based authentication code must be used  5-inform the patient about who use the data, the purpose of the collection, the privacy methods used, the rights they have and a contact method in PP.  6- The retention policy should be included in the PP.  7- In case of a PHI breach, the competent authority and the user must be notified |
| Olff (36) | 2015 | To present academic challenges in developing and evaluating m-Health tools | App review | trauma and posttraumatic  stress disorder apps | Developments in m-Health tools are reviewed with examples from the field of trauma and posttraumatic stress disorder, and challenges are presented. | 1-Access control  2- Security and confidentiality  3- Data storage  4-data ownership | 1-No unauthorized access is allowed  2- appropriate collection and handling of user data  3- Safe storage of the material collected.  4- Who has access to the data and who owns the data should be addressed. |
| Plachkinova et al. (37) | 2015 | To propose a taxonomy incorporating the most significant security and privacy aspects of mHealth applications. | Propose a testing method | General | They developed the  Taxonomy using the security challenges in a mobile healthcare environment, the threat taxonomy for mHealth privacy, and classified the types of mHealth apps proposed in other studies. Then they assessed 38 apps using their taxonomy. | 1-Security  1-1-Authentication and Authorization (AA);  1-2-Integrity and Accountability (IA);  1-3-Ease of Use and Availability (EUA);  1-4-Confidentiality, Management, and Physical Security  2- Privacy  2-1-Identity threats  2-2-Access threats  2-3-Disclosure threats | 1-1-Authentication: distinguish legitimate users from imposters. Authorization: a particular user has the right to carry out a certain activity  1-2- Accountability: answer to the appropriate authority. Integrity: Information may be altered when it is exchanged in an insecure network.  1-3- Users do not want complex security that will slow down their tasks. Information is available only authorized individuals.  1-4- Properly managed to ensure the normal flow of operation and information involved. Ensure the security of the mobile device as well as the back-end datacenter.  2-1-Protect patients’ identity  2-2- Patients should have ultimate control on the collection, use, and disclosure of PHI  2-3- Comply with HIPAA and HITECH |
| Sunyaev et al. (56) | 2015 | To assess mHealth apps for the presence and scope of PPs, and what information they offer | Mhealth Apps assessment | Medical and Health & Fitness | They checked the PPs on the app store web site for the particular app. | PP coverage  1-Type of information  2-Rationale for collection  3-Sharing of information  4-User controls | 1-Whether information (operational, behavioral, and sensitive) is collected  2-inform user about app operation, Personalization, and secondary use of data  3- whether information is shared for service provision, social interaction, with third parties  4-user’s ability for supervision, correction, and notification (about changes to PP) |
| Scott et al. (19) | 2015 | To identify a set of risk and safety features for evaluating mHealth apps | Mhealth Apps assessment | General | 20 most popular mHealth apps were assessed using a set of risk and safety features determined through literature search | 1-User Registration  2-Data Storage  3-Sharing of Information  4- Authentication  5-Manipulate Data  6-Inform user about privacy/security measures  7-PP | 1-It is consumers’ responsibility whether or not to provide information to health apps  2-Data are stored locally or remotely  3-Data are shared with a 3rd party/ advertiser  4-To guarantee the privacy of users’ data  5-Enable users to update, correct, and delete their data  6- To building the trust required  7-Existence of privacy policy |
| Bruggemann et al. (39) | 2016 | To conduct a feasibility study and develop a prototypical  instantiation of an information privacy risk index for mHealth apps | Propose a testing method | Medical and Health & Fitness | They identified information privacy risk factors through reading the description of the apps and inspecting the screenshots offered in the app store; then they assessed all apps. | 1-Information Sharing targets  2-Types Information  3- User authentication  4-Connection Security  5-Unspecificc Information Transfer  6-Reasonable Information Collection | 1-target or host destination to which apps send users' personal information  2- Type of information collected  3-requiring login information  4-use encryption  5- whether apps used click tracking analytics tools or contacted advertisement  servers to display advertisement banners  6- reasonableness of collection of personal information |
| Chen et al. (40) | 2016 | Present an application that manages  securely personal health information on a mobile platform | Propose a secure app | General | The proposed app is a one stop center designed for individuals and doctors to store and update an individual medical record into an anonymous database  as well as to retrieve personal information. | 1-Incognito  2Access Control  3-Privacy Control  4-Authentication  5-Encryption  6-Multifactor Authentication  7-Emergency Control | 1-The centralized server stores the records in incognito form by discarding all identifiers of the records  2-user registration and control the access to the records, and the personal sharing list  3-allow patients to control the access level of their health information by third parties  4-verify the identity of patient and doctor using a unique Master ID and a secret key  5- The master ID will be encrypted with the doctor’s secret key.  6- to provide a multi-layered defense authentication so that it will be more difficult for unauthorized access  7-the ability to activate emergency module. |
| Jones and Moffitt (41) | 2016 | To examine and review the existing  federal policies, APA guidelines,  industry’s mobile app development guidelines, and to provide recommendations | Examine ethical guideline | Mental health apps | This study discusses the methods in which app developers and providers can safeguard against violations of privacy and confidentiality | Authorized access  Breach notification  Data backup  Encryption  Informed consent  Manage Widgets  Access control  User authentication  Safe data Transfer | Prevent unauthorized access  Inform users when their unprotected information has been breached  Information be kept offsite (i.e., a cloud server) in the case of a disaster or an electronic version stored on multiple backups  Minimum-security requirements for using servers (i.e., such as having at least a 256-bit encryption)  obtain user consent in Terms and Conditions for enabling notifications, and what is displayed on the screen of their mobile  customizable, to protect the user’s privacy  who has access to the information collected and to what level?  User name and password  Transfer data using Bluetooth or a data cable; Cloud servers should be used only for backup and not for transferring information |
| Loy et al. (42) (Singapore) | 2016 | To develop a quality assessment tool for evaluating apps  and assess the quality of such apps | Propose a testing method | Apps for medication-related problems | A quality assessment tool consisted of 4 sections (appropriateness, reliability, usability,  privacy), for evaluating medical apps  were developed 59 apps were included to assess their quality. | 1-Privacy existence  2-Choose sharing content  3- Choose who to share content with | PP should mention the collection of user data and how it is being used  Users should be able to choose what content to share, and to select a person whom they want to share content with |
| Mense et al. (43) | 2016 | To presents the results of the technical analysis of  mHealth applications running on Android regarding privacy and security risks. | Mhealth Apps assessment | Health and fitness | They assessed the security of the apps in a specific test environment, and a proxy which worked like a “man in the middle attack” | User permissions  Communication (share) with the developer-controlled website  Communication with third party (advertising, analytics) sites  PP | Users had to grant a broad range of permissions  Use encrypted (SSL) communication; HTTPS protocol it makes it harder to intercept data – but not impossible.  communication with the advertising sites should be encrypted  Users should be informed about what data are collected, stored, shared, or used by whom. |
| Morera et al. (44) | 2016 | To presents a guide regarding security solution | Propose a guideline | “medicine” or “health and wellbeing” | After including apps, the most widespread  weaknesses in the field of security in the development of these  mobile apps were examined | 1-Server-Side Controls  2-Data Storage  3-Transport Layer Protection  4-Data Leakage  5-Authorization and  Authentication  6-Cryptography  7-Client-Side Injection  8-Security Decisions via  Untrusted Inputs  9-Session Handling  10-Binary Protections | 1-The hostile data can trick the interpreter into executing unintentional commands and access unauthorized data.  2- lost or stolen mobile devices, or any malicious codes which circumvent any encoded information  3- protect network traffic by strong coding  4- How applications interact with all the elements of the device which are not the property of the developers.  5- apply authentication and session management are frequently correctly  6-strong modern encryption/ decryption  7- internal users or the application itself can send untrusted data to the system  8- allow communication with other trusted  applications, sensitive operations should require interaction with the user  9- handle the session correctly once it is opened  10- Lack of binary protection allows an attack through reverse engineering. |
| Asaddok & Ghazali (45) | 2017 | To design and propose a usability, security  and privacy taxonomy | Propose a testing method | General | They propose a usability, security and privacy taxonomy based on a similar study, and then evaluated the propose taxonomy by conducting some sessions with a focus group | 1-Security   - 1. Confidentiality   2. Integrity   3. Availability   2-Privacy  2-1- Identity  2-2- Access  2-3- Disclosure | 1-1-allow only authorized access to information  1-2-use secure network to prevent Information alteration  1-3-Loss of availability can have severe consequences for users who rely on the app for decision-making.  2-1-patients losing/sharing their identity credentials, enabling authorized access.  2-2- patients have ultimate control on the collection, use, and disclosure of PHI.  2-3- secure data transmission; prevent disclosure of data about the location or sensor type of the patients. |
| Wu et al. (46) | 2016 | To offer a summary  of current knowledge, practices, and existing gaps regarding confidentiality and privacy | Viewpoint paper | Child and  Adolescent Psychiatry apps | This article presents a summary of current confidentiality and  privacy issues regarding services provided by Child and  Adolescent Psychiatry apps and provides a discussion on how the field can respond to this new challenge. | 1-Disclosure of information sharing  2-Access privilege  3-Privacy and trust | 1-Disclosure about who will be able to access data from a mental health app; disclosure in the setting of emergencies: the thresholds/ types of entered data that may potentially activate an emergency response system.  2-Authentication and login procedures for parents and patients;  Design different levels of access to restrict sharing of confidential health information with parents or guardians; likewise, allow the parents the option of keeping sensitive family histories confidential  Data security  3-PP provide information about data sharing/usage (sold, traded, or marketed); Updated privacy laws on deidentified data, recording and transmitting data,  regular security enhancements |
| Grindrod et al. (20) | 2017 | To explore the privacy and security | Mhealth Apps assessment | Medication management. | After including apps, using a standard data collection form, 2 reviewers independently coded each app for the presence/absence of the predetermined security and privacy criteria. | 1-Security  1-1-Password protection available  1-2-Medication alerts available  2-Privacy  2-1-Type of information collected  2-2-PP | 1-1- log in using a mandatory email and password combination  1-2- Private alerts (e.g., hidden from lock screen)  2-1- personal information, contact information and health information.  2-2-inform users about data collection, encryption, remote wipe and third-party sharing. |
| Gabel et al. (47) | 2018 | To realize privacy by design by privacy engineering methodologies in the context of an mHealth project and the accompanying studies. | Propose a privacy scenario | General | Privacy requirements of the scenario presented in this study are modelled based on methodologies as privacy protection goals and privacy design strategies. | 1-Privacy:  1-1-Unlinkability  1-2-Transparency  1-3-Intervenability  1-4-Confidentiality  1-5-Availability  1-6-Integrity | 1-1-Minimize the collection/processing of identifiable data; hide strategy: Hide: (Strong pseudonymization techniques); Separate: use a distributed pseudonym; Abstract: the collected data  1-2-inform users about data usage and potential data breaches,  1-3-allow the user to control personal data processing; role-based access control.  1-4-encryption to ensure confidentiality  1-5-Availability of the server & backup  1-6-Integrity of communication and data via TLS |
| Hussain, et al. (48) | 2018 | To propose and evaluate a framework to improve the security of medical data | Mhealth Apps assessment | General | A sample of 100 mHealth apps was used to check how the proposed security framework works against leakage of private information, and what steps it takes to prevent this leakage. | 1-Authentication  2-Encryption  3-Privacy Terms & Conditions  4-Prevent Leakage of  Information  5-Suitability of requested data  6-Use external devices | 1-To enforcing confidentiality, integrity, and availability.  2-Use encryption through https rather than the use of http.  3-Existence of PP  4-To prevent leakage of information, the “malware-checker” is asked to examine the app against malware behavior.  5-To understand the requested permissions and how a combination of some requested permissions can misuse medical information  6,7-Using external devices to enhance the functionality of the phone would impose serious threats to users’ data such as device MisBonding attacks and privilege escalation attacks |
| Papageorgiou et al. (49) | 2018 | To provide an in-depth security and privacy analysis of  apps | Mhealth Apps assessment | General | They performed both static and dynamic analysis of selected mhealth apps, along with tailored testing of each application's functionalities. | 1-Privacy  1-1-PP  1-2-Permissions  2-Security  2-1-App logs  2-2-random number generators  2-3-encryption  2-4-external storage  2-5-secure WebView  2-6-secure transmission  2-7-share with 3^rd^ parties | 1-1-accessible up to date PP;  1-2-ask normal permissions and provide justification fir that  2-1-sensetive information should not be logged  2-2-use secure random number generators  2-3-sensetive information like (password, keys, etc.) should not be stored on files; information should be encrypted  2-4-apps should not be able to read/write external storage  2-5-secure implementation of WebView and SSL  2-6-store health-related data locally or transmit over HTTPS connections  2-7-wether information is shared with 3^rd^ parties |
| Scott et al.  (50) | 2018 | To explore international  regulation of health apps and existing standards for app developers | Mhealth Apps assessment | maternal & child health apps | A set of assessment criteria was developed based on a literature review and then the trustworthiness of the included apps was evaluated. | 1-Access permission  2-Privacy policy | 1-Determine the level of access required by the app to the user’s device/data/ content.  2-Developed a legally binding PP that adheres to the content of the app; Where the app developer is extracting data, and how this information is being used. |
| Zelmer et al. (51) | 2018 | To develop guiding  principles and criteria for a framework | Propose an assessment framework | mental health apps | A set of guiding principles and criteria was developed | 1-Transparency of Information Security  2-Information Security  3-Transparent PP | 1-The app’s security and privacy policies should be transparent and easy to find.  2-The app should meet minimal standards for information security and privacy.  3-Is the PP transparent and easy to find. |
| Minen et al. (13) | 2018 | To assess whether there are privacy issues surrounding apps | Mhealth Apps assessment | Headache and migraine apps | 29 apps were examined using a developed database which assess the types of data the apps requested for input by the user and whether the apps had clear privacy policies. | 1-Collected data  2-Registration/Log In  3-Data storage  4-Law and regulations  5-PP content | 1-what personal information the app collects  2-if the app has any Registration/Log In available  3-whether data are stored locally or remotely, store data on the phones rather than to app company’s own servers  4- regulation and which country they belong to  5-type of information collected, data sharing, the purpose of data sharing, protection of minors, data access, and user rights, user ability to correct or delete data. |
| Al-Sharo (52) | 2019 | To analyze the status of privacy and security in relation to mhealth | Review | General | The analysis or the review has been done through academic literature review, a study of the laws which regulate mobile health in the EU and USA. | 1-Authentication System  2-Encryption Technique  3-Data storage secure techniques  4-Transmission of data security  5-Privacy and security laws, regulation  6-Privacy and security in the mHealth emergency system  7-Users’ consent  8-Location privacy  9-PP | 1-Authentication System  2-Encryption Technique  3-Data storage secure techniques  4-Transmission of data security  5-Privacy and security laws  6-Privacy and security in the mHealth emergency system  7-Users’ consent  8-Location privacy  9-PP |
| Huckvale et al. (14) | 2019 | To provide a contemporary assessment of the privacy practices of popular apps | Mhealth Apps assessment | Depression and smoking cessation apps | PP content of 36 top-ranked apps was evaluated with prespecified criteria. Technical assessment of encrypted and unencrypted data transmission was performed. | 1-1-Privacy policy content:  Primary/Secondary uses of collected data  Data sending/sharing  Procedures for opting out of online data sharing  Consequences of not providing or sharing data  Asserting non-identifiable data collection only  data retention  Inherent risks or limitations of security  use cookies  access requests  manipulate data by developer or 3rd parties  Complaints procedures  procedures for vulnerable or at-risk users  Legal jurisdictions policy  Date of policy/ next review  Procedures for changing the terms of the policy  Procedures after takeover  Identity of responsible legal entity  2-Technical and procedural security arrangements security | 1-Primary/Secondary uses of collected data; Data/Sending sharing; Procedures for opting out of online data sharing; Consequences of not providing or sharing data; Asserting non-identifiable data collection only;  How long data will be retained; Inherent risks or limitations of security using public internet; How cookies will be used; Procedures for subject access requests; Procedures for editing/deleting data held by developers or third parties; Complaints procedures; Special procedures for vulnerable or at-risk users and/or children; Identity of data controller or responsible legal entity; Legal jurisdictions policy/ governing data processing; Date of policy/ next review; Procedures for changing the terms of the policy; Procedures after takeover or dissolution of legally responsible body  2-eg, anonymization, Secure Sockets Layer, secure servers, limited access, backup |
| Iwaya et al.  (53) | 2019 | To present a Privacy Impact Assessment for mHealth Data Collection Systems (MDCSs) | Propose a privacy framework | General | A Privacy Impact Assessment for MDCSs is presented based on the EGDPR, providing a systematic identification and  evaluation of potential privacy risks. | 1-Privacy Principles  1-1-Quality of data processing  1-2- Processing lawfulness (and informed consent)  1-3-Information right of data subject  1-4-Access right of data subject  1-5-Intervenability  1-6- Data subject’s right to object  2-Security of processing  3- Accountability | 1-1-Ensuring processing in a lawful, fair, and transparent manner, only for legitimate purposes; Providing purpose specification; Ensuring limited processing for specified purpose; Ensuring data avoidance/ minimization/quality, accuracy, and integrity; Ensuring limited storage  1-2- Ensuring legitimacy of personal data processing  1-3-Providing adequate information in cases of direct collection of data from the data subject or where data has not been obtained directly from the data subject (eg, from third parties)  1-4-Facilitating the provision of information about processed data and purpose/ an (electronic) copy of data  1-5-Facilitating the rectification, erasure, or blocking of data/ portability of data/ the notification to third parties about rectification, erasure, and blocking of data; Providing the ability to withdraw consent  1-6- Facilitating the objection to the processing of personal data/ direct marketing activities/ disclosure of data to 3rd parties/decisions that are solely based on automated processing of data/ Facilitating the data subject’s right to dispute the correctness of machine conclusions  2-Ensuring the confidentiality, integrity, and availability of personal data storage, processing, and transmission; Ensuring the detection of personal data breaches and their communication to data subjects  3-Ensuring the accountability of personal data storage, processing, and transmission |
| Müthing et al. (54) | 2019 | To identify relevant security concerns on the server side of mHealth apps, test a mHealth apps, and compare the servers used by mHealth apps with servers used in all domains | Mhealth Apps assessment | General | Server security characteristics relevant to the security of mHealth apps were assessed, presented, and discussed. To evaluate servers, appropriate tools were selected. 60 apps were selected and tested. | Security  1-1-Use of secured connections (SSL/TLS)  1-2-SSL/TLS version  1-3-Key exchange support  1-4-Cipher support  1-5-Certificates  1-6-Vulnerabilities  1-7-Hypertext Transfer Protocol Strict Transport Security | 1-1- The use of any unsecured connections  1-2- Evaluating the supported versions of SSL/TLS  1-3- The cryptographic algorithm used to exchange the keys during the handshake for the following symmetric encryption  1-4-The cipher negotiated between client and server dictates what symmetric encryption is applied after the handshake and key exchange  1-5- The security characteristics TLS offers rely on the server’s certificate. Any trust issues here are critical  1-6- Certain attacks are based on specific implementations or the absence of a patch on the server  1-7- Support HSTS can prevent downgrades to HTTP |
| Nurgalieva et al. (55) | 2020 | To examines  the recent literature on security and privacy of m-Health applications | Review | General | This study analyzed data security and privacy evaluation techniques and frameworks that have been proposed for mHealth applications | 1-data security  2-data integrity  3-data encryption  4-user authentication  5-transport security  6-secure data storage  7-pp  8-Data ownership  9-Permission system as auditability  10-data anonymization  11-data retention  12-data access mechanism | 1-data security  2-data integrity  3-data encryption  4-user authentication  5-transport security  6-secure data storage  7-pp  8-Data ownership  9-Permission system as auditability  10-data anonymization  11-data retention  12-data access mechanism |
