# Supplementary material for: Critical Criteria and Countermeasures for Mobile Health Developers to Ensure Mobile Health Privacy and Security: Mixed Methods Study
Source: JMIR Mhealth Uhealth. 2023 Mar 2;11:e39055. doi: 10.2196/39055 (PMC10020905; doi:10.2196/39055)
Supplement: Multimedia Appendix 3 [file mhealth_v11i1e39055_app3.docx]

**Multimedia appendix 3**. **The** **complete list of removed criteria according to the different phases of the study**

| **Removed criteria** | **Phase** |
| --- | --- |
| Are the terms of use of the cloud services clearly stated? | After calculating impact scores |
| Are there appropriate technical measures to control access? | After calculating impact scores |
| Does the application have the ability to be deleted remotely? | After calculating impact scores |
| Is the application able to read/write external storage? | After calculating impact scores |
| Does the privacy policy explain the procedures for vulnerable or at-risk users? | After calculating impact scores |
| Is there an explanation about the application inherent risks or limitations of security? | After calculating CVR |
| Is data file obtained from insecure sources, offline, and with limited permissions stored? | After calculating CVR |
| Is there a process in place to create, register, review and modify the access? | After calculating CVR |
| Does the application use a cipher suite? | After calculating CVI |
| Does the application use binary code to be protected against attacks through reverse engineering? | After calculating CVI |
| Does the application ensure legitimacy of personal health data processing? | After calculating CVI |
| Is an accurate and recoverable copy of stored health information when required before moving instrument? | After calculating CVI |
| Are the internal users or the application itself able to send untrusted data to the system? | After calculating CVI |
| Does the application consider a strategy to guarantee the availability of the server and backup for users who rely on the app for decision-making? | After calculating CVI |
| Does the application minimize the collection/processing of identifiable data? | After calculating CVI |
| Does the application obtain an informed consent prior to using the user's personal information? | After calculating CVI |
| Does the application provide a secure key exchange? | After calculating CVI |
| Is the audit conducted to properly control harmful activities and ensure that they do not occur? | After calculating CVI |
| Does the application use pseudonymisation techniques? | After calculating CVI |
| Are emergency measures taken to continue the processes that are critical to maintaining the security of health information? | After calculating CVI |
| Does the application have the ability to activate emergency module? | After expert panel |
| Does the application use secure Android WebViews? | After expert panel |
| Does the application warn the users about losing/sharing their identity credentials which enables authorized access? | After expert panel |
| Does the application use the proper session handling while service transactions between the mobile app and the server? | After expert panel |
| Does the privacy policy explain the procedures after takeover or dissolution of legally responsible body? | After expert panel |
| Is the application able to manage widget to protect the user’s privacy? | After expert panel |
